# Semantic Adaptive Communication Based on Double-Attention Phase and Compress Estimator for Wireless Image Transmission

**DOI:** 10.3390/s25237201

**Published:** 2025-11-25

**Authors:** Hong Yang, Lijuan Wang, Pingyu Wang, Ji Li, Linbo Qing, Xiaohai He

**Affiliations:** 1 College of Electronics and Information Engineering, Sichuan University, Chengdu 610065, China; yhscu@scu.edu.cn (H.Y.); wanglijuan@stu.scu.edu.cn (L.W.); liji@stu.scu.edu.cn (J.L.); qing_lb@scu.edu.cn (L.Q.); hxh@scu.edu.cn (X.H.); 2 Tianfu Jiangxi Laboratory, Chengdu 641419, China

**Keywords:** adaptive communication, semantics communication, attention mechanism, image transmission, wireless transmission

## Abstract

In existing semantic communication systems for image transmission, some images are generally reconstructed with considerably low quality and a high transmission rate. Driven by the imperative to effectively tackle these longstanding challenges, semantic communication has emerged as a critical technological advancement. In this work, we propose a Semantic Adaptive Communication (SAC) framework to transmit images with core information. Specifically, the proposed framework is composed of a Semantic Encoder (SE) and Semantic Decoder (SD), a Semantic Code Generator/Restore (SCG/SCR) module, a Compression Estimator (CE), a Channel State Information Acquisition (CSIA) module and a Wireless Channel. To fully capture both channel attention and spatial attention for semantic features, we design a Double-Attention Module (DAM) that operates alongside channel conditions, integrated into the SE and SD. Additionally, in order to predict the compress rate of the SAC, the CE works based on the channel condition and the recover quality. The experimental results demonstrate that the proposed SAC framework has a greater PSNR (increased by 0.5–2 dB) and accuracy value (91–93%), which indicate the SAC robustness, than traditional communication methods and other semantic communication algorithms in image transmission scenarios. In addition, the proposed framework achieves adaptive transmission rates with minimal sacrifice in recovery performance while enhancing the bandwidth utilization efficiency of the semantic communication system.

## 1. Introduction

With the rapid advancement of the Internet of Things (IoT), Wireless Sensors Networks (WSNs), artificial intelligence (AI), and 6G communication technologies, communication technology is undergoing unprecedented transformations—shifting beyond the constraints of traditional data protocol frameworks. Recently, emerging semantic communication (SC) technology is breaking through the limitations of traditional systems, protocols, and networks. It focuses on addressing the challenge of cross-platform interoperability, which enables intelligent interactions, including machine-to-machine and human-to-machine communication. Therefore, this technology also lays a solid foundation for the future vision of the “intelligent connectivity of all things” [1].

In recent years, the theoretical research of the SC has further driven the evolution of related communication frameworks and accelerated the advancement of communication technologies. At the very beginning, Kountouris et al. [2] provide a clear definition and detailed elaboration of an SC system module. Interestingly, Convolutional Neural Networks (CNNs) possess the inherent capability to more effectively capture local features in images through their receptive field mechanisms [3]. This unique advantage provides a novel research perspective for integrating SC with deep learning (DL) [4,5], aiming to extract high-level semantic information from images while mitigating the limitations of traditional pixel-level transmission. Instead, it achieves the most efficient transmission under existing channel conditions and task requirements with stronger robustness and anti-interference capabilities.

### 1.1. Artificial Intelligent Wireless Transmission

In the AI wireless transmission field, Mohammad et al. [6] proposed an edge-native intelligence approach for 6G communication, leveraging the power of federated learning (FL). The authors provided an overview of the FL algorithm and its recent advancements, exploring potential implementations in various beyond-5G (B5G)/6G wireless networks. Additionally, the article identified several future research opportunities arising from emerging technologies and highlighted the challenges associated with the operation of FL in future wireless networks. Additionally, deep learning (DL), a sub-field of machine learning, has garnered significant attention in wireless communication networks. Chen et al. [7] have presented how artificial neural network (ANN)-based ML algorithms can be employed for solving various wireless networking problems. Their comprehensive tutorial highlights the integration of intelligent, data-driven functions across the wireless core and edge infrastructure to enhance ultra-reliable low-latency communications and IoT connectivity. Similarly, Zhang et al. [8] conducted a thorough survey on DL in mobile and wireless networking, providing an extensive overview of various DL techniques and their potential applications in mobile and wireless contexts. Notably, they discussed both the advantages and challenges involved in integrating DL into wireless communication networks. In an alternative work, Zhang et al. [9] proposed a new communication paradigm called the intellicise communication system with model-driven SeC. This paradigm enables the communication system to evolve from the traditional transmission of bits to the transmission of models. The authors concluded that this model-based approach is a new feature of joint source–channel coding (JSCC). Further, they analyzed the performance evaluation metrics and the implementation details of the intellicise communication system. Note that most of the existing works on the combination of AI and wireless networks use AI to improve the performance of a single module or aspect, such as channel coding, signal detection, and networking, while the intent and meaning of the source are not considered. In comparison, SeC delves into the semantic level of the source and changes the basic way of information interaction, which drives an integrated enhancement of the overall communication systems and networks. Furthermore, AI is a pivotal technology for these two categories due to its powerful capabilities of self-learning and non-linear representation, which help in solving communication issues, such as the transmission optimal representation of semantic information and adaptive transmission strategies.

Image reconstruction is one of the most important applications of semantic communication in computer vision tasks. In semantic communication tasks, the receiver not only needs to reconstruct the pixel distribution of the transmitted image, but also maintain semantic consistency to meet specific task requirements. Therefore, image reconstruction applications in semantic communication enable transmitted information to be more compact and task-oriented. Task-oriented semantic communication has garnered extensive research attention [10]. For example, researchers have designed DL-based end-to-end image semantic communication systems [11], which transmit only the key semantic information of images. This research demonstrates that semantic communication achieves higher communication efficiency compared to traditional communication paradigms. The classification tasks can be completed by quantifying semantics and then jointly transmitting text or image information through source-channel coding [12]. In autonomous driving scenarios, a semantic communication system has been developed with image segmentation as its task backdrop [13]. By transmitting only the semantic information relevant to image segmentation, this system successfully achieves the goal of reducing bandwidth consumption. In addition, to optimize resource utilization, researchers have proposed a resource allocation method [14]. This method enables dynamical adjustment of network resources while achieving low latency and high accuracy in semantic communication processes.

### 1.2. Semantic Communication

By exploring the transition from traditional data rate maximization to meaning-centric communication, Lan et al. [15] delineated the SeC framework alongside technological advancements pertinent or beneficial to SeC. Then, they explored the knowledge graphs (KG)-based SeC technologies and applications for various scenarios. Moreover, according to the 6G visions, potential technologies and use cases that are helpful for SeC are introduced. Additionally, Luo et al. [16] delved into the fundamentals of SeC, examining its principles, architecture, and potential to transform intelligent applications in 6G networks. Especially, they highlighted the role of SeC in addressing the challenges posed by traditional communication systems when supported the high data requirements of future intelligent services. Complementing these insights, Yang et al. [17] emphasized the motivations and strong justifications for integrating SeC into 6G. The authors presented an overview of research in this field and discussed three major types of SeC, including semantic-oriented, target-oriented, and semantic-aware communication. Zhang et al. [18] proposed a predictive and adaptive PADC framework, which enables flexible rate optimization and meets the given target transmission quality requirements. In an alternative work, Zhang et al. [19] provided a brief review of SeC, presenting potential use cases and identifying open challenges. In this work, the authors introduced a new semantic representation framework, namely semantic base (Seb), and established an intelligent and efficient semantic communication (IE-SC) network architecture. The proposed architecture integrated AI and network technologies to enable intelligent interactions among various communication objects. Furthering the discourse, Trevlakis et al. [20] presented a semantic networking architecture that leveraged point-to-point SeC systems for goal-specific semantic information extraction and filtering. This architecture aims to transform communication into a multi-user, distributed, edge-to-cloud network. They introduced various application scenarios of SeC, the challenges of semantic noise, and the complexity of implementing advanced network infrastructure. A more recent study conducted by Lu et al. [21] presented a comprehensive taxonomy of SeC research, including theories, applications, metrics, and implementations. This work not only synthesized the current landscape but also charted future directions, underscoring SeC’s potential in enhancing the efficiency, reliability, and intelligence of wireless systems.

**Figure 1:**
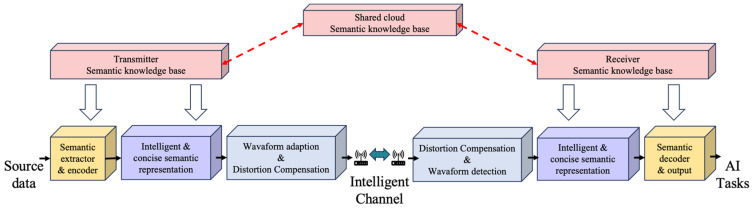
The structure of an SeC architecture.

The collective research into SeC architectures (as shown in Figure 1) and applications marks a significant evolution towards meaning-centric communication. These studies highlight SeC’s crucial role in enhancing network efficiency, reliability, and intelligence. The exploration of innovative network architectures opens new pathways for addressing contemporary communication challenges and setting the stage for future advancements.

### 1.3. The Challenges in SeC

To address the challenges of limited bandwidth and low data transmission efficiency, researchers have proposed a wireless image transmission system based on semantic communication [22]. This system can effectively reduce bandwidth requirements while maintaining high image quality during transmission. To tackle the challenge of channel conditions impairing image reconstruction quality, adversarial training is employed to incorporate semantic information into the training set. In this way, this approach effectively mitigates the interference of channel noise on a system’s transmission performance [23]. Yang et al. [24] propose a transformer-based wireless image semantic transmission system, which enhances both bandwidth utilization efficiency and image reconstruction quality. Notably, it achieves favorable reconstruction results even under extremely limited bandwidth conditions, making it particularly suitable for image transmission in smart network environments.

In essence, channel adaptation pertains to channel condition and the bandwidth constraints, while key metrics such as Signal-to-Noise Ratio (SNR) and the compression ratio (CR) are associated with transmitted data. Since each SC module is trained under a specific SNR or CR condition, multiple SC modules are trained to cover all operational regimes of the SNR and CR channels. Only when the SNR or CR values of a channel are close to their corresponding training parameters can the modules achieve optimal performance in wireless image transmission tasks. Moreover, even with a sufficient number of SC modules trained under diverse channel parameter configurations, an adaptive rate control for specific image transmission tasks with quality constraints remains unachievable. This challenge stems from the inability to predetermine the quality of the recovered image prior to transmission. For these reasons, as an emerging methodology for wireless image transmission, the proposed SC modules proposed above also suffer from two key challenges: the SNR adaptation and the CR adaptation.

To address these two challenges, Xu et al. [25] propose a Deep Joint Source–Channel Coding (Deep JSCC) module with an integrated attention module. This module enables an adaptive adjustment of the learned image features under varying channel SNR conditions. The JSCC framework [26] draws inspiration from the CR and channel coding rate optimization methods. It incorporates a Squeeze-and-Excitation (SE) module [27] to adjust adaptive learned image features under varying SNR conditions, thereby maximizing image reconstruction quality. In order to achieve adaptive rate control capability for wireless image transmission, Yang et al. [28] proposed a Deep JSCC method. Zhang et al. [18] proposed a Variable Deep Joint Source–Channel Coding (VDJSCC). Furthermore, for task requirements, Yang et al. [29] proposed a scalable semantic adaptive communication.

However, in the current semantic communication (SC) module, transmission quality is jointly influenced by channel conditions, code rates, and data content. Furthermore, the relationship among these three factors is highly complex and difficult to formulate explicitly [18]. Therefore, in addition to addressing the SNR and CR adaptation challenges, a robust SC module must also tackle the problem of transmission quality prediction and adaptation adjustment. This capability enables the transmitter to achieve high-quality, rate-guaranteed transmission for each piece of semantic information.

### 1.4. The Contribution of Our Work

To tackle the aforementioned challenges, this paper proposes a Semantic Adaptive Communication (SAC) framework based on a CNN-transformer network for wireless image transmission. This framework achieves adaptation adjustment and operational flexibility in response to varying SNR, CR, and image content. The primary contributions of this paper are outlined as follows:

(1) To enable flexible adaptation of Semantic Encoders and Decoders amid dynamic channel condition changes or across varying SNR values, this paper overcomes the key limitation of existing modules—most of which rely on fixed architectures tailored to a single SNR scenario. Specifically, we design an SAC framework comprising a lightweight Semantic Encoder (SE) and Decoder (SD), Semantic Code Generator (SCG), Semantic Content Restore (SCR) mechanism, and a Compression Estimator (CE), which generates flexibly adjustable semantic information to adaptive transmission.
(2) To achieve the minimum CR value within a specific range of SNR for the CR adaptation, this paper designs a CR prediction module termed Compression Estimator (CE) via local decoding quality and channel conditions. This module reduces training complexity and time costs, as it only requires a single training process for intelligent recovery. Furthermore, by lever aging decoded image information, it also enhances the efficiency of image reconstruction tasks.
(3) To enhance image reconstruction quality under constrained channel conditions, this paper designs an SE and SD module based on residual blocks, incorporating considerations of optimal feature selection and reconstruction accuracy. These components integrate a channel condition aware Double-Attention Module (DAM) so as to effectively strengthen the expressive capability of key semantic information and to improve module robustness, thus meeting the high demand for detail preservation and structural restoration in image reconstruction tasks. Furthermore, the proposed module achieves cross-data reconstruction generalization and can deliver high-quality image reconstruction results by adapting to diverse task requirements and dynamic channel conditions.

The remainder of this paper is structured as follows: Section 2 elaborates on the framework of the proposed SAC; Section 3 presents the simulation results and corresponding analysis of the proposed method; and finally, Section 4 summarizes the key conclusion of this paper.

## 2. Proposed Semantic Adaptive Communication Framework

**Figure 2:**
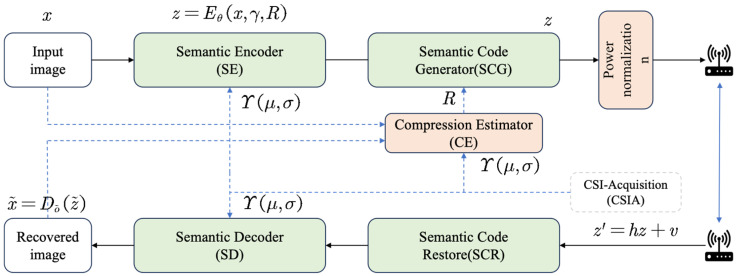
Overview of the proposed SAC framework.

The entire SAC framework, as shown in Figure 2, continues to adopt an end-to-end training paradigm to ensure its ability to adapt to dynamically varying channel conditions, thereby achieving optimal image reconstruction performance across diverse channel scenarios.

With wireless network transmission protocols [30], the CSIA module enables the acquisition of channel fading matrix h and SNR. These critical channel parameters are then fed into other modules, directly supporting the operations of the SE, SD, and CE. The workflow of this communication framework is described as follows:

① First, raw images $x\in R^{d_{in}}$ are fed into the SE module $E_{\theta }$, where $d_{in}$ denotes the dimension of the input image. This process yields semantic features with specific dimensions, denoted as $x_{\alpha }$. Then, these semantic features are encoded into complex-valued semantic feature codes $x_{c}\in C^{k}$ via the SCM module. Finally, contextual semantic features z are computed by the SE module based on the SNR as follows:

(1) $$z=E_{\theta }(x,\gamma ,R)$$

where γ denotes the channel condition SNR and R represents the semantic compression ratio of the SCM with the custom input compression ratio range from 0.05 to 0.5.

② The semantic features are first normalized and then transmitted over the Wireless Channel as follows:

(2) $$z^{\prime }=hz+v$$

where h∈C denotes the channel gain. $v\in CN(0,\omega^{2}I)$ represents additive Gaussian White Noise, where CN denotes the additive Gaussian distribution and $\omega^{2}$ is the noise power. If neglecting the influence of channel gain for simplification, it can be rewritten as follows:

(3) $$z^{\prime }=z+v$$

③ Subsequently, the transmitted signal is received and decoded by a semantic decoder *D_δ_* to obtain a decoded image as follows:

(4) $$\overset{\sim }{x}=D_{\delta }(\overset{\sim }{z})$$

### 2.1. Semantic Encoder and Decoder

This paper fully combines the advantages of Convolutional Neural Networks (CNNs) and the ability of a transformer to capture local and long dependencies. Specifically, a hybrid encoding structure is adopted to optimize transmission performance, thereby meeting the requirements of intelligent tasks for image data transmission in semantic communication scenarios.

**Figure 3:**
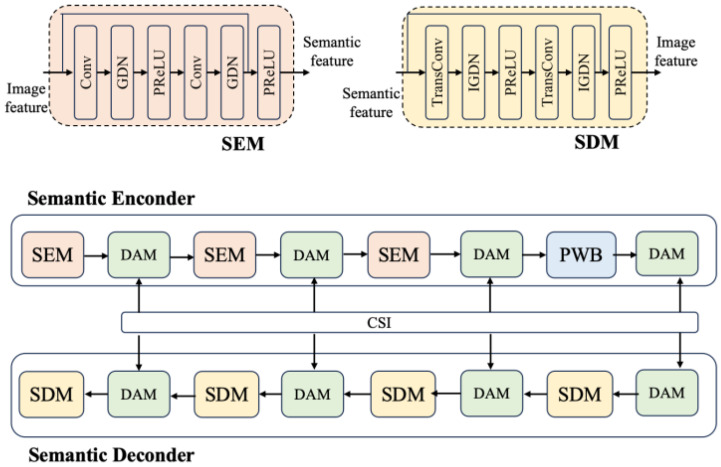
Structure of SE and SD.

As depicted in Figure 3, the SE comprises three Semantic Encoder Component (SEC) and four Double-Attention Module (DAM) units, modulated based on distinct channel condition, and one Partial Window Block (PWB). The PWB is employed solely as a residual structure, rather than the commonly used down-sampling structure. It inherits only a portion of the Swin Transformer architecture, which helps reduce module complexity in the context of semantic communication and artificial intelligence tasks.

The SEC focuses on extracting local semantic features of images, while the PWB module learns global semantic features. Meanwhile, the decoder is aimed at restoring reconstructed images with the same size as the original images, thus being structurally symmetrical to the encoder. It consists of four SDMs and four DAMs, but without the PWB.

#### 2.1.1. The Partial Window Block (PWB)

The PWB is designed by adopting a Window Multi-head Self-Attention (W-MSA) module from the improved version of the Partial Window Transformer (PWT) [31]. It can enhance multi-scale feature extraction capability, improve module representational power, and strengthen performance in intelligent tasks within semantic communication systems. In addition, in order to reduce the complexity of the SAC, the PWB deletes the Shifted Window Multi-head Self-Attention (SW-MSA) module.

#### 2.1.2. The Double-Attention Module (DAM) Based on Channel Condition

In the field of SeC, existing deep learning modules often struggle to effectively adapt to dynamic changes in channel conditions. The CSI modulation feature block, implemented within a CNN framework, aims to dynamically adjust semantic features. In addition, CSI is represented by SNR to enhance the module’s robustness of time-varying channel environments. When training the SAC framework, the input SNR and the compression rate are randomly sampled for each batch, ensuring variability in SNR and compression rate across batches.

**Figure 4:**
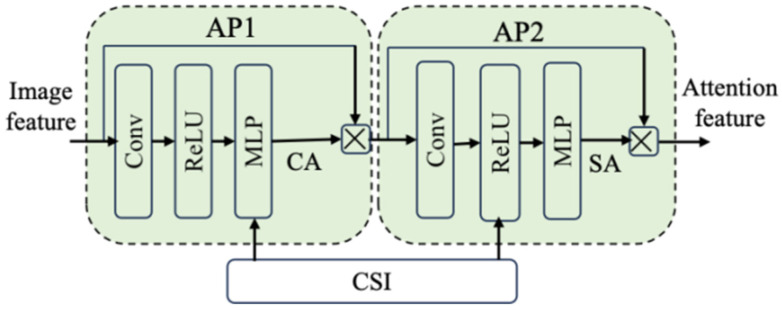
The structure of DAM.

**Figure 5:**
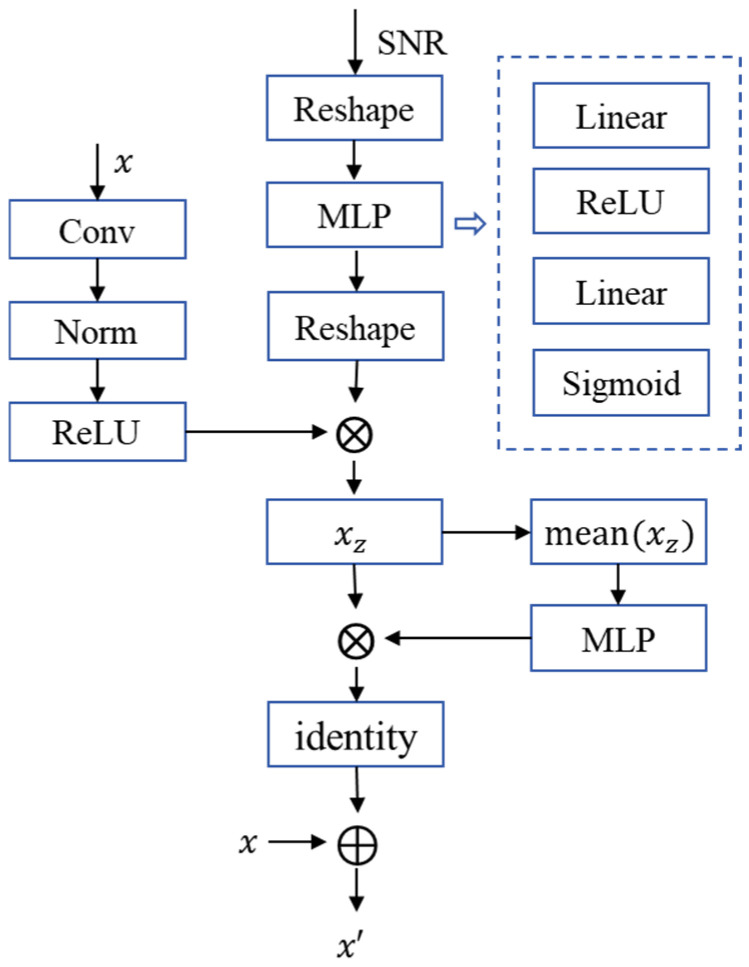
The structure of AP in the DAM.

As illustrated in Figure 4, we propose a Double-Attention Module (DAM), which can capture both channel attention (CA) and spatial attention (SA), and is based on integrating convolution, non-linear transformation, and an SNR-adaptive mechanism. This module enables the adaptive adjustment of semantic attention features, thus improving the robustness of the entire semantic communication system. As shown in Figure 5, the two Attention Phases (APs) in the DAM share a similar structure. Their workflow is as follows:

① Firstly, the module takes encoded semantic features and channel condition SNR as inputs. The semantic features *x* undergo local information extraction and generate residual information *x*’ with the same size as the original semantic features x, thereby preserving the original features’ information.

② Next, the SNR dimension is extended to match the semantic features x, enabling the module to learn the impact of the channel on features. A SNR adjustment factor is generated via a Multi-Layer Perceptron (MLP).

③ Then it is multiplied with the semantic features x through an element-wise product to enhance semantic features $x_{z}$ using SNR information. Subsequently, the enhanced semantic features $x_{z}$ are averaged and processed by an MLP to generate channel adjustment factors. These factors are applied via element-wise multiplication to achieve channel enhancement and residual information *x*’ generation.

④ Finally, the input features are fused with the residual information *x*’ to obtain SNR-modulated semantic features, thereby adapting to the semantic transmission requirements under varying channel conditions.

(5) $$x^{\prime }=x⊕fac[mean(x_{Z})]$$

### 2.2. The Semantic Code Generator (SCG) and Semantic Content Restore (SCR)

**Figure 6:**
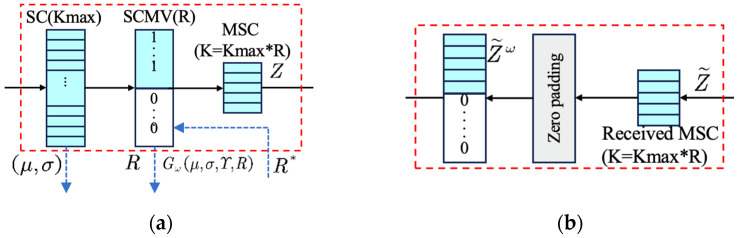
The structure of (**a**) SCG and (**b**) SCR.

As shown in Figure 6, the semantic features S unfolded by the Semantic Encoder are represented as $F\in R^{d_{in}\times C}$, where $d_{in}$ denotes the spatial dimension of the semantic features and C denotes the number of channels, R* indicates the estimated required compression ratio for this time; these features are then fed into the semantic feature compression module and masked using binary SCM vectors (SCMV) for semantic features processing. Ultimately, the compressed semantic features can be formulated as follows:

(6) $$F^{\prime }=[S\cdot R]$$

where [·] represents the rounding operation and $R\in {\left\{0,1\right\}}^{d_{in}\times C}$ is used to selectively activate or block specific semantic features. Specifically, a value of 1 indicates transmitting the corresponding semantic feature, while a value of 0 means ignoring it. The total number of semantic features to be transmitted, denoted as $F^{\prime }$, is determined by the compression ratio R. This mechanism ultimately enables transmission with adaptive adjustment of semantic features.

### 2.3. Compression Estimator (CE)

**Figure 7:**
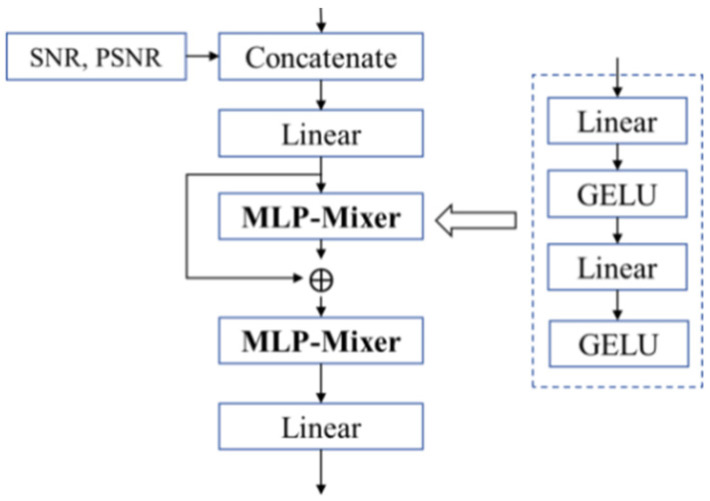
The structure of CE.

To predict the minimum transmission rate for image transmission in wireless image transmission systems, this paper draws inspiration from the *PSNR* prediction network framework in [18]. It proposes an image compression rate *R* estimation algorithm based on semantic features *f_s_* and the correlation between the reconstructed image *PSNR* and the channel condition SNR. Accordingly, a Compression Estimator (CE) is designed to leverage statistical features and channel information to match the reconstruction task. As shown in Figure 7, the working principle of a CE network is described as follows:

① First, image statistical features *f_s_*, *SNR*, and *PSNR* are input separately. The image statistical features include mean and standard deviation, which describe image brightness, contrast, and local variations. Specifically, the mean characterizes the overall brightness of an image, while the standard deviation quantifies image contrast and can approximately reflect the impact of channel conditions on image quality without relying on complete image data. SNR serves as a metric for transmission channel conditions, and *PSNR* is used to measure image information loss. *CON* means the fusion method. Thus, the CE network can determine the minimum compression rate of output images under varying channel conditions *SNR* and *PSNR* values without requiring a full encoding–decoding module. It further predicts the minimum compression rate R under a target *PSNR*, thereby enhancing semantic communication performance and task execution efficiency.

② Next, the input features are mapped to hidden dimensions via a linear layer, followed by residual information generation and fusion through an MLP-Mixer layer. Feature fusion is performed using another MLP-Mixer layer. The MLP-Mixer structure employs linear layers, GELU activation functions, and other operations for compression rate prediction. It primarily conducts features and information exchange between samples, improves the stability of model training, accelerates convergence, and enhances the model’s anti-over fitting capability.

③ Ultimately, the CE achieves a prediction of the required compression rate *R* for image transmission based on *SNR* and *PSNR* values, providing a reference for transmission decisions.

(7) $$R=f_{s}⊕CON(SNR,\;and\;PSNR)$$

## 3. The Training of the Proposed Framework

### 3.1. Model Optimization

According to the task requirements of the SAC framework, the training objective is to minimize the transmission overhead of semantic features while minimizing the reconstruction loss between the reconstructed image $\overset{\sim }{X}$ and the original image X (measured by mean square error). The training process is detailed as follows:

① Input processed image data $X\in R^{C\times H\times W}$, where C,H,W denote the number of channels, height, and width of the image, respectively.

② The image is processed by a Semantic Encoder $E_{\theta }(\cdot )$ with an attention mechanism to extract SNR-weighted semantic features $F=E_{\theta }(X;\gamma )\in R^{d}$ in specific dimensions. Subsequently, using the semantic compression mask vector SCMV, the semantic features are compressed at a given semantic compression rate to obtain the compressed semantic code $F^{\prime }=g_{r}(F)$, where $F^{\prime }=C^{K}$.

③ Normalize the power of $F^{\prime }$ and generate Z for transmission over the wireless physical channel.

④ The receiver acquires noise-affected semantic features $z^{\prime }$ and decodes them via a joint source–channel decoder $D_{\delta }(\cdot )$ to reconstruct the image as $\overset{\sim }{X}=D_{\delta }(z^{\prime })$.

When training the SAC framework, the input SNR is configured as γ∈[0, 27] dB, and the compression rate is set to r∈[0.05, 0.5]. During training, these two parameters are randomly sampled for each batch, ensuring variability in *SNR* and compression rate across batches. As the number of training samples increases, the SAC framework automatically optimizes the end-to-end source-channel joint coding process, learning optimal performance under diverse SNR and compression rate conditions. Table 1 illustrates a single training round of the SAC framework.

### 3.2. Semantic Loss Function

This paper focuses on the task of image reconstruction, so its semantic loss function differs from that used in image classification tasks. In image classification, the most commonly employed loss function is Cross Entropy Loss (CEL). In contrast, for image reconstruction tasks, mean squared error (MSE) can intuitively quantify the accuracy of image restoration and is therefore widely adopted as a loss function to evaluate the performance of models in reconstructed image tasks. The objective of this paper is to minimize the difference in *PSNR* between the reconstructed image and the original image. Specifically, the MSE loss function calculates the pixel-level discrepancy between the original image and the reconstructed image, defined as follows:

(8) $$MSE=\frac{1}{N}\sum_{i=1}^{N}(x_{i}-{\overset{\sim }{x}}_{i}{)}^{2}$$

where $x_{i}$ is the ith pixel value of the original image, ${\overset{\sim }{x}}_{i}$ represents the ith pixel value of the reconstructed image, and N represents the total number of pixels in the image.

However, the MSE loss function only measures pixel-level errors and fails to accurately reflect human visual perception of image quality. Therefore, this paper integrates the Peak PSNR into the model reconstruction quality evaluation algorithm to assess the overall image quality. Specifically, the combined use of *MSE* and *PSNR* enables a comprehensive evaluation of the model performance for image reconstruction tasks. Mathematically, the calculation formula for PSNR is as follows:

(9) $$PSNR=10\cdot \log_{10}(\frac{MAX_{I}^{2}}{MSE})$$

where $MAX_{I}$ denotes the maximum possible pixel value of the image. For the 8-bit images used in experiments (with pixel values ranging from 0 to 255), $MAX_{I}=255$. For images normalized to the range [0,1], $MAX_{I}=1$. Here, MSE represents the mean squared error.

## 4. Experiment

### 4.1. Experimentation Details

All experiments of the SAC framework mentioned in this paper were verified and simulated in the Linux system, and the experimental parameters are shown in Table 2.

The parameters of the SAC framework are updated using the Adam optimizer with adaptive learning rate adjustment. The mean square error (MSE) is adopted as the loss function to guide module learning through quantization error minimization. After each training epoch, the model is tested on the test set to evaluate its reconstruction performance. When the model achieves the minimum loss value on the test set, its parameters are saved. Training is terminated and the final parameters are retained upon reaching the predefined number of training iterations. The hyper parameters of the SAC model are configured as follows: a total of 400 training epochs, an image batch size of 128, and an overall model learning rate of 0.001. For the CE model, only the number of epochs needs to be set to 150, while other parameters are same as those of the SAC model. Detailed parameters are provided in Table 3.

### 4.2. Datasets

This section of experiments focuses on two key first verification objectives. First, it verifies the reconstruction performance of the proposed SAC framework. Second, it validates the framework’s generalization and adaptive capability across different datasets. To demonstrate the effectiveness of the proposed algorithms, the experiment is designed with a specific dataset setting. The model is trained exclusively on CIFAR-10 and STL-10. However, it can be tested on multiple datasets by loading the pretrain weights. These testing datasets include CIFAR-10, CIFAR-100, STL-10, and Kodak. Thus, the training datasets used in the experiment are CIFAR-10 and STL-10, while the testing datasets are CIFAR-10, CIFAR-100, STL-10, and Kodak.

The CIFAR-10 dataset contains 100 classes, with a total of 60,000 images. Thus, the number of samples per class is only 600, including 500 for the training set and 100 for the testing set. In contrast, the CIFAR-100 dataset has more classes with fewer images per class. This characteristic makes CIFAR-100 more challenging than CIFAR-10 and better suited to demonstrate the generalization capability of the SAC framework. Additionally, both the STL-10 and Kodak datasets are larger-scale datasets with higher resolution images. Selecting diverse datasets enables more comprehensive validation of the SAC framework’s reconstruction adaptability.

### 4.3. Comparison with State-of-the-Art Methods

Experimental results are evaluated and demonstrated using comparative methods such as quantitative visualization and qualitative functional analysis. Since the SAC framework in this paper focuses on image reconstruction tasks, five comparative simulation experiments are designed to compare its performance with that of the Deep JSCC-V [18] and traditional VAE-JSCC methods [32], ADJSCC [25], Diff-JSCC [33], and SGD-JSCC [34], as shown in Table 4.

### 4.4. Ablation Study

We conduct a series of experiments to validate the adaptive compression rate, rate predictably, and robustness under varying channel conditions of the proposed SAC framework:

① To evaluate the reconstruction performance of the proposed adaptive model, a compression rate and channel adaptability test was conducted, and then the classification accuracy performance is evaluated.

② A data adaptability test is performed under the condition of disjoint training and testing datasets, validating the generalization capability of the SAC framework for handling complex tasks.

③ It is verified that the proposed CE model can predict the compression rate of the SAC framework with minimal error.

④ The model complexity is assessed using metrics such as floating-point operations (FLOPs), module parameter count, and memory usage.

#### 4.4.1. Different Compression Ratios

Within a specified SNR range, we train the proposed SAC framework and Deep JSCC-V model [18]. Subsequently, the trained model weights are applied to the testing dataset to verify their performance in image reconstruction tasks. The two models are evaluated for their reconstruction performance over AWGN and Rayleigh channels, respectively. The SNR training range is set to [0, 27] dB, and the compression ratio training range is configured as [0.05, 0.5]. The reconstruction quality of publicly available datasets (CIFAR-10 and STL-10) is assessed, with the experimental evaluation metric being PSNR. In each training iteration, the test network randomly samples different channel conditions and compression rates within the predefined SNR and R-train ranges. This ensures that the obtained experimental results are comprehensive and can fully validate the reconstruction performance of the two models across diverse channel environments. In contrast, the traditional VAE method only performs training and testing under a fixed SNR and without compression.

To further compare the image reconstruction performance between the proposed SAC framework and the Deep JSCC-V algorithm [18], this experiment configures the test compression rate (R_test) as [1/16, 1/12, 1/6, 1,3]. These values correspond to low, medium, high, and ultra-high compression rates, respectively. Additionally, the robustness of the two algorithms under diverse channel conditions for image reconstruction tasks is further evaluated, and compared with the traditional VAE method.

**Figure 8:**
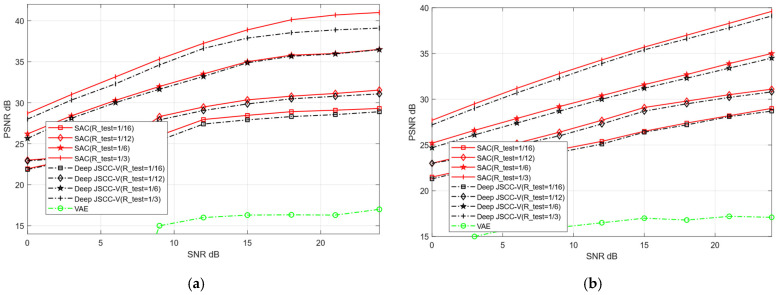
Comparison with Deep JSCC-V [18] of CIFAR-10 (**a**) AWGN, (**b**) Rayleigh.

**Figure 9:**
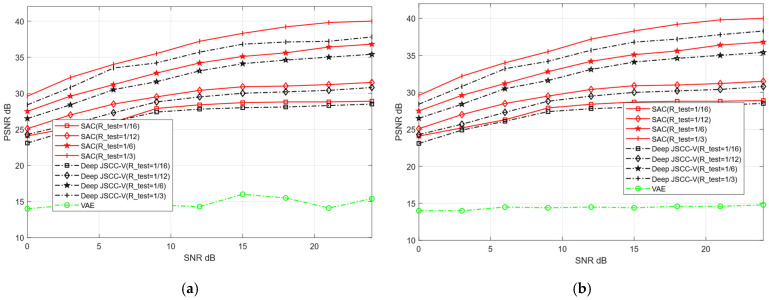
Comparison with Deep JSCC-V [18] of STL-10 (**a**) AWGN, (**b**) Rayleigh.

Figure 8 and Figure 9 illustrate the image reconstruction performance of different models under AWGN and Rayleigh channels, as well as across varying compression rates. In addition, it can be observed that the proposed SAC framework achieves higher PSNR values for reconstructed images compared to the Deep JSCC-V algorithm [18] and the traditional VAE method, regardless of the compression ratio and channel condition. This demonstrates its significant advantages in image compression and reconstruction. Meanwhile, the SAC framework attains optimal reconstruction quality under diverse channel conditions, indicating stronger robustness. As the compression ratio increases, the PSNR of both the SAC framework and Deep JSCC-V algorithm [18] continues to rise, suggesting that the SAC framework can deliver superior reconstruction quality in resource-constrained scenarios.

These results collectively indicate that the SAC framework preserves more image details during compression and reconstruction. This advantage stems from its improved encoding structure and dynamic features adjustment mechanism based on the DAM, enabling the SAC framework to adapt to varying compression rates and channel conditions. Consequently, compared to the Deep JSCC-V algorithm [18], the SAC framework exhibits higher efficiency in feature extraction, semantic information compression, and adaptive selection, while the former can achieve more accurate image reconstruction.

**Figure 10:**
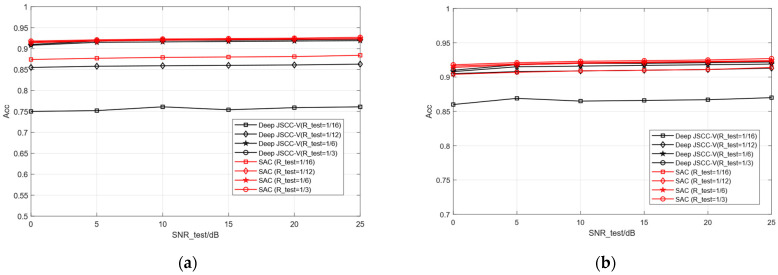
Comparison of classification accuracy of Deep JSCC-V [18]. (**a**) STL-10 test images, (**b**) CIFAR-10 test images.

At this point, the classification performance of each is studied based solely on the different lengths of semantic features (i.e., different compression rates), as shown in Figure 10. In the case of a single model, the overall performance of the proposed SAC model is equivalent to or even better than that of multiple models trained by Deep JSCC -V using different compression rates. This indicates that SSAC can achieve scalable semantic features by adapting to different compression requirements through a single training session, thereby helping to reduce the resource consumption of training and deployment. This is highly attractive for application scenarios that require the deployment of efficient semantic communication systems in resource constrained environments

#### 4.4.2. Cross-Dataset Adaptive Reconstruction

To verify the adaptive reconstruction capability of the proposed SAC framework across diverse datasets, this experiment designates CIFAR-10 and STL-10 as training sets, and CIFAR-100 and Kodak as testing datasets for image reconstruction tasks. Training on relatively simple datasets enables the model to rapidly learn fundamental image features. In contrast, testing on complex and sensitive datasets serves to validate the model’s generalization ability in handling intricate image reconstruction scenarios. The training parameters of both models remain consistent with the previous experiment, with only the test set replaced.

**Figure 11:**
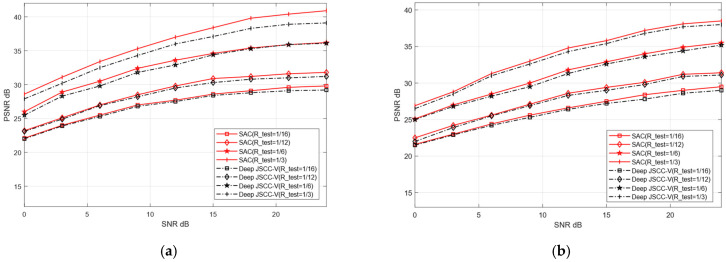
Comparison of Deep JSCC-V [18] from CIFAR-10 to CIFAR-100 (**a**) AWGN, (**b**) Rayleigh.

**Figure 12:**
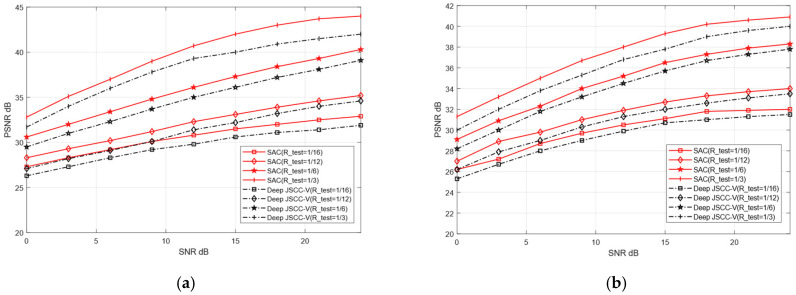
Comparison of Deep JSCC-V [18] from STL-10 to Kodak (**a**) AWGN, (**b**) Rayleigh.

Although training and testing sets originate from distinct datasets, this may cause reconstruction performance degradation. As shown in Figure 11 and Figure 12, when the test dataset is switched from CIFAR-10 to CIFAR-100 and from STL-10 to Kodak, the reconstruction PSNR of the SAC framework does not decline significantly. This indicates the SAC framework can robustly adapt to more complex images. Even under cross-dataset training test splits, it shows such adaptability. In addition, Figure 10 and Figure 11 further demonstrate that the SAC framework outperforms Deep JSCC-V [18] in reconstruction performance. This is across diverse channel conditions. Additionally, as the compression ratio increased from 1/16 to 1/3, the SAC framework exhibited stronger data adaptability than the Deep JSCC-V algorithm [18]. This advantage becomes increasingly prominent for large-sized images, highlighting the SAC framework’s superiority in complex image reconstruction scenarios.

These results indicate that, despite differences in data sources between the training and testing sets, the proposed SAC framework can still maintain relatively high reconstruction quality and exhibits stronger robustness in data-adaptive reconstruction. This robustness may stem from the SAC framework’s enhanced effectiveness in extracting and preserving valuable semantic features during the compression and recovery processes, thereby ensuring robust and superior reconstruction performance when handling diverse dataset types.

#### 4.4.3. Compression Estimator

**Figure 13:**
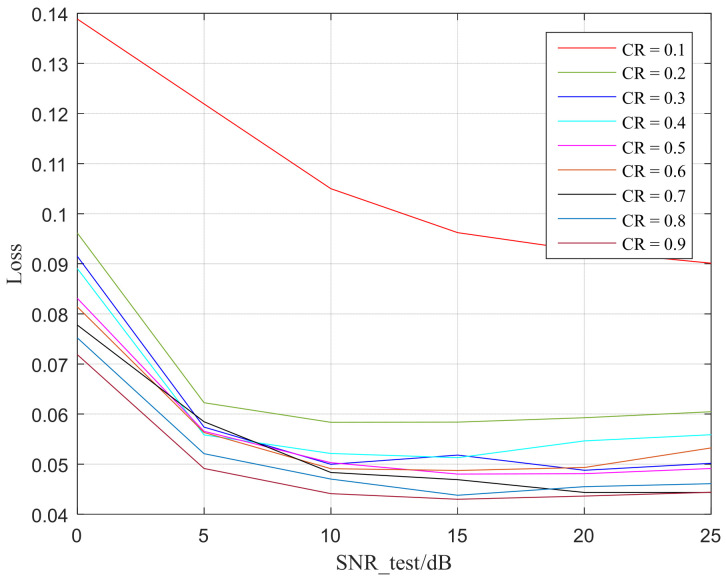
Loss of CE predicted.

The predictive characteristics of different objects and images is explained as a feature of randomly selected images showing a gradually decreasing trend from low to high channel numbers, indicating that, after learning, the semantic features of low and high channels have more information compared to each other [29]. In order to indicate the loss coming from the channel condition and different CR, from Figure 13 can be observed that, at extremely low SNR, such as 0 dB, the CE model predicts relatively high losses. This is because, at very low SNR, semantic features are relatively more damaged by noise in the AWGN channel, resulting in significant errors between the predicted classification results and the actual classification results.

**Figure 14:**
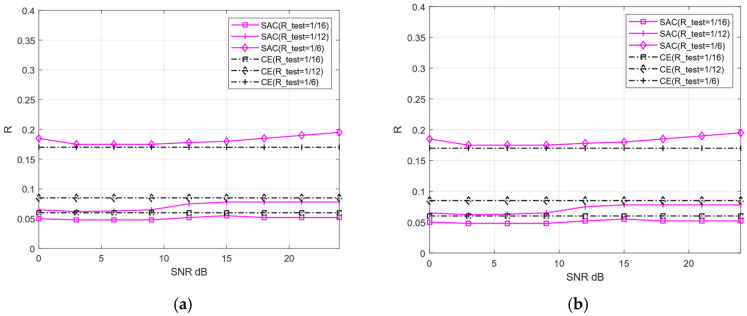
The comparison of predicted and actual compression rates of CE (**a**) AWGN, (**b**) Rayleigh.

To verify the compression rate predictive performance of the proposed CE, we separate the AWGN and Rayleigh channels to derive prediction models. Subsequently, the predicted compression rates are compared against the actual compression rates required by the SAC framework under varying compression ratios and channel conditions. Figure 14 documents the predicted versus actual compression rates of the CE under compression ratios of 1/16, 1/12, 1/6, and 1/3, as well as SNR spanning [0, 27] dB.

Figure 14 reveals that the actual compression ratio values across different SNR levels are not significantly different from the compression ratio predicted by the CE, with a maximum discrepancy of approximately 0.02. Intuitively, compression ratio prediction accuracy should improve as the SNR increases. However, the opposite trend emerges at high SNR (e.g., exceeding 15 dB). This phenomenon arises because the reconstruction performance of the SAC framework tends to converge once the SNR reaches a certain threshold. Even with a further increase in SNR, the model’s reconstruction quality changes minimally, which introduces greater uncertainty into the CE prediction model and results in a slight increase in prediction deviations. Overall, CE exhibits small prediction errors, validating its effectiveness in compression ratio prediction.

#### 4.4.4. Model Complexity

The SAC framework proposed in this paper comprises an SE, SD, SCG, CE, and Wireless Channel. Specifically, the SE is constructed with an SEM and a DAM block, which adapt to different PSNR and PWT conditions. The SD, on the other hand, is composed of an SDM and a DAP. These modules collectively ensure superior reconstruction quality and robustness. This experiment evaluated the model complexity by measuring three key metrics: FLOPs, Parac, and memory usage within the algorithm. As presented in Table 5, it can be observed that the model achieves high-quality reconstruction for semantic communication tasks while maintaining low complexity. Notably, the algorithm within this model exhibits a low FLOP count, which accelerates the training speed of the SAC framework. From these data, it can be seen that the SAC framework in this article still has advantages in resource-limited wireless communication scenarios.

### 4.5. Visualized Analysis

**Figure 15:**
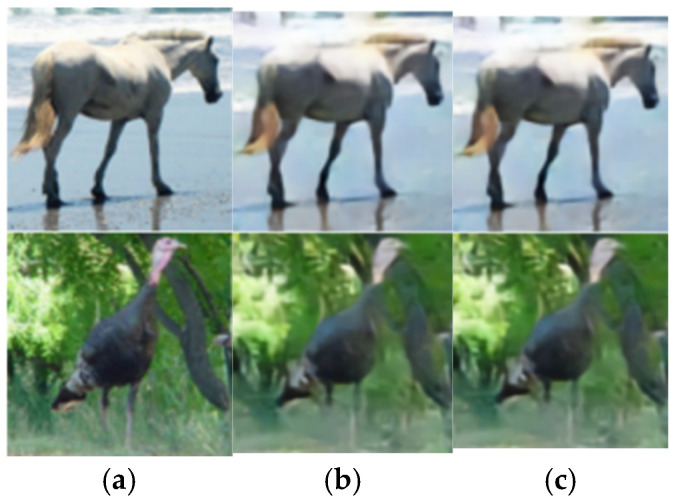
Visualization results of cross-dataset reconstruction under AWGN channel (**a**) the original image (**b**) R_test = 1/12, SNR_test = 3 dB (**c**) R_test = 1/3, SNR_test = 24 dB.

To further demonstrate the image reconstruction performance of the SAC framework, extensive experiments are configured with SNR set to 3 dB and 24 dB, and compression rates R set to 1/12 and 1/3, respectively. As shown in Figure 15, we analyze the visualization results of reconstructed frames for four images from the STL-10 dataset. The image reconstruction outcomes under an AWGN channel can be seen. Comparative analysis reveals that, at SNR = 3 dB and R = 1/12, higher channel noise and stronger compression lead to degraded reconstruction quality with significant detail loss. Despite these challenges, the SAC framework retains core structural information. Conversely, at SNR = 24 dB and R = 1/3, lower noise and milder compression enable higher-quality reconstruction with finer detail restoration, resulting in a greater similarity between the reconstructed and original image. This visual comparison of SAC’s performance across varying SNRs and compression ratios further validates the model’s robustness and effectiveness in semantic communication-driven image reconstruction.

Based on all the experimental results, it can be concluded that, under a specific compression ratio and SNR constraints, the proposed SAC framework exhibits strong adaptability to diverse channel environments and maintains favorable image reconstruction performance even at high compression rates. This validates the model’s robustness. Additionally, this paper introduces a compression rate prediction network that can accurately estimate the minimum compression ratio required for image reconstruction, thereby reducing model complexity and enhancing communication efficiency.

## 5. Conclusions

The SAC framework in this paper consists of SE, SD, SCG, CE, and Wireless Channel. It realizes quality prediction and adaptation for SNR and CR values. The SCG module achieves variable semantic length and predicts compression rate through CE, endowing encoding results with greater flexibility. Moreover, a DAM block is introduced to weight-extracted features, dynamically adjusting their importance. This optimizes the compression and restoration processes of images, effectively preserving image detailed information and enhancing reconstruction quality. Furthermore, the compression rate prediction network CE, based on statistical features and channel conditions, enables fast and accurate estimation of the compression rate for image reconstruction. Experimental results demonstrate that the SAC framework ensures high quality image reconstruction under limited transmission resources and exhibits strong robustness under low-SNR conditions. The experimental results demonstrate that the proposed SAC framework has greater PSNR (increased by 0.5–2 dB) and accuracy value (91–93%) than traditional communication methods and other semantic communication algorithms in image transmission scenarios, which indicates the SAC robustness. Especially, the proposed SAC scheme has the advantage in resource-limited wireless communication scenarios.

## Figures and Tables

**Table 1:** The training steps of the proposed SAC framework.

One Round of Training Step	
Input: dataset of images X to be reconstructed, Batch size B, learning rate η;	
Output: SAC framework parameters (θ,δ);	
1:	Sample a batch of data $x=[x_{,},\ldots ,x_{B}]$ from the dataset;
2:	Perform the following operations on each data sample $x_{j}$ in x;
3:	Randomly generate channel signal-to-noise ratio $\gamma_{j}$;
4:	Randomly generate compression ratio $r_{j}$, $r_{j}\in [0.05,\;0.5]$;
5:	Input the image samples $x_{j}$ to be reconstructed into the encoder network to obtain the encoded semantic features ${x^{\prime }}_{j}$;
6:	The semantic feature compression $z_{j}$ calculation is obtained through Equation (1);
7:	Randomly generate physical channel noise $\gamma_{j}$ based on SNR v;
8:	Obtained $z_{\;j}^{\prime }$ through Equation (2);
9:	Input the noised semantic features $z_{\;j}^{\prime }$ into the decoder network to obtain the reconstructed image ${\overset{\sim }{x}}_{j}$;
10:	End the operation for each sample;
11:	Calculate mean square error loss: $L_{\theta ,\delta }=\frac{1}{B}\sum_{i=1}^{B}{\left(x_{j}-{\overset{\sim }{x}}_{j}\right)}^{2}$
12:	Update the module parameters (φ,θ).

**Table 2:** The experimental environment of the proposed SAC.

Hardware/Dataset	Description
CPU	Intel® Core^TM^ i7-9700
GPU	NVIDIA GeForce RTX 4060 Ti
Memory Capacity	32 GB
GPU Driver Version	535.146.02
CUDA Version	11.7
Operating System	Ubuntu 18.04.6 LTS
Network	1000 Mbps

**Table 3:** The training parameters of the SAC framework.

Parameters	Values
Epochs (SAC)	400
Epochs (CE)	150
Batch Size	128
Optimizer	Adam
Learning Rate (LR)	0.001
Loss Function	MSE
SNR	[0, 27] dB
Compression Ratio (CR)	[0.05, 0.5]

**Table 4:** The comparison of our SAC framework with existing framework.

Parameters	R = 1/3 (Compression Rate), SNR = 5 dB (Channel Condition)	
	AWGN	Rayleigh
Deep JSCC-V [18]	23.7 dB	22.5 dB
VAE-JSCC [32]	22.1 dB	10.4 dB
ADJSCC [25]	24.8 dB	20.2 dB
Diff-JSCC [33]	22.0 dB	19.7 dB
SGD-JSCC [34]	23.5 dB	19.0 dB
SAC Framework (ours)	24.1 dB	23.2 dB

**Table 5:** The Complexity of module.

Module	FLOPs	Parac	Memory
Deep JSCC-V [18]	4.92 G	63.79 M	48.84 MB
SAC	2.98 G	38.51 M	28.98 MB

## Data Availability

The research data in this paper is private and cannot be disclosed.
